# Microdissection of Human Esophagogastric Junction Wall with Phase-contrast X-ray CT Imaging

**DOI:** 10.1038/srep13831

**Published:** 2015-09-08

**Authors:** Jianfa Zhang, Guangzhao Zhou, Dongping Tian, Runhua Lin, Guanyun Peng, Min Su

**Affiliations:** 1 First Affiliated Hospital of Shantou University Medical College, Shantou, Guangdong, People’s Republic of China; 2 Shanghai Institute of Applied Physics, Chinese Academy of Sciences, Shanghai 201204, China; 3 Institute of Clinical Pathology & Department of Pathology, Shantou University Medical College, Shantou, Guangdong, People’s Republic of China; 4 The Judicial Critical Center, Shantou University Medical College, Shantou, Guangdong, People’s Republic of China

## Abstract

Phase-contrast x-ray imaging using an x-ray interferometer has great potential to reveal the structures inside soft tissues, because the sensitivity of this method to hydrogen, carbon, nitrogen, and oxygen is about 1000 times higher than that of the absorption-contrast x-ray method. In this study, we used phase-contrast X-ray CT to investigate human resected esophagogastric junction. This technology revealed the three-layer structure of the esophagogastric junction wall—mucous, submucosa and muscular layers. The mucous and muscular layers were clearly separated by a loose submucosa layer with a honeycomb appearance. The shape of the mucous and muscular layers was intact. The boundary between the mucous and submucosa layers was distinct, as was the border of the muscular and submucosa layers. The surface of the esophagogastric junction was displayed clearly through 3D reconstruction. The technology might be helpful in the diagnosis of esophagogastric junction lesion, especially for the early adenocarcinoma.

The esophagogastric junction (OGJ) is located between the oesophagus and the stomach. It is a highly specialized anatomical region that is often difficult to understand^1^. The esophagogastric junction is important that backflow of stomach secretions into the oesophagus is controlled at the OGJ opening only transiently to allow passage of the swallowed food into the stomach. The diaphragmatic hiatus, through which the oesophagus passes at the OGJ, has a role in this valvular mechanism^2^,^3^. Its dynamic action is dependant upon the intrinsic and extrinsic effects of its own anatomical structure and its position with respect to the surrounding organs in this region of the body^4^.

Several structures are important in maintaining a barrier at the OGJ. The lower oesophageal sphincter (LOS) forms part of the OGJ structure. The intrinsic muscles of the distal oesophagus and the sling fibers of the proximal stomach make up the internal mechanism structures of the LOS. The muscles of the diaphragm hat connect to the OGJ make up the crural diaphragm and this constitutes the external LOS mechanism structures. The tissue that connects the distal oesophagus to the crural diaphragm is known as the phreno-oesophageal ligament^5^.

The OGJ can be distinguished from the body of the oesophagus by its behavior pattern. There is an increase in the tone of the circular muscle in this region. The sphincter relaxes in response to a swallow and this usually precedes the arrival of a contraction wave travelling down the oesophagus. This phase of relaxation is followed by a short-lasting elevation of pressure above resting values. In recent years it has been known that the sphincter sometimes relaxes even when a swallow does not occur. These relaxations are known as transient lower oesophageal sphincter relaxations (tLOSRs)^6^. Pressures in the abdominal and thoracic cavities are involved in creating the barrier, as well as the exact intra-abdominal location of the junction. Time and posture are also important factors. Various kinds of diseases, including malignant tumors, occur frequently in the esophagogastric junction. The adenocarcinoma incidence of the OGJ has obviously increased in recent years. However, the prognosis of patients with adenocarcinoma of esophagogastric junction (AEG) has not been improved, because of the result of major patients being diagnosed at an advanced stage^7^. Therefore, there is a significant need for improved non-invasive, high-resolution imaging techniques that can display clearly in the esophagogastric junction wall for the early detection and diagnosis of esophagogastric junction adenocarcinoma.

The esophagogastric junction examination mainly depends on these methods such as CT^8^,^9^,^10^,^11^, magnetic resonance imaging (MRI)^10^,^12^, endoscopy^13^ and gas-barium doublecontrast X-ray gastrointestinalgraphy^14^.

Gas-barium double contrast X-ray gastrointestinalgraphy is a common clinical tool to assess esophagogastric junction conditions. CO2-barium is most commonly used, because of higher safety and lower price. Before the examination, some aerogenic powders are taken orally by patients. Reactions between the dry powders won’t take place until they encounter water. The esophagogastric junction will be expanded by the produced CO2 gas. After a few minutes, the patients take orally barium. The CO2-barium double contrast X-ray imaging can not only provide optimal visualization of mucosal fold, but also evaluate the esophagogastric junction peristaltic function^14^. However, the method is prohibited to use, when a patient is suspected of gastrointestinal perforation or complete obstruction. Because barium sulfate leaking out of the digestive tract can cause infection or granuloma formation for gastrointestinal perforation patients, while gastrointestinal obstruction in patients with oral barium sulfate may enhance obstruction symptoms. So, iodine is usually used in these patients. Moreover, conventional X-ray double-contrast imaging involves absorption imaging, which distinguishes different tissues according to the linear attenuation coefficient (μ). The μ values are proportional to tissue density. With 1- to 2-mm spatial resolution with conventional imaging, the lowest density difference that can be detected is only 0.01 g cm^−3^. Hence, although high contrast can be achieved for tissues with different proportions of light and heavy elements, distinguishing the subtle structure differences in soft tissues that contain only light elements is difficult. The low-density contrast of X-ray absorption imaging is the main obstacle in clinical practice^15^.

Clinical CT provides good spatial resolution while MRI provides good contrast resolution of soft tissues. They may clearly show the esophagogastric junction relationship with adjacent tissue (iodine is usually used in order to enhance contrast of gastrointestinal tract with the surrounding tissue in CT scan). However, both of the techniques can’t display fine structure of esophagogastric junction wall due to their limited image resolution being on the millimeter-scale. In addition, MRI has limited spatial resolution and long acquisition time^16^,^17^. The micro-CT can distinguish a tissue density difference of 0.01 g/cm3. The spatial resolution of micro-CT can reach 2 μm^18^, but its image resolution is still at millimeter-scale if without the use of imaging agents. Endoscopy is the only technique that can be used to directly observe the esophagogastric junction lumen *in vivo*, the esophagogastric junction mucosal folds are clearly observed. However, the downside of endoscopy is invasiveness and may cause discomfort even the risk of perforation and bleeding for patients during examination. In addition, the technique has limitations in discerning esophagogastric junction wall layering.

Synchrotron-radiation hard X-ray phase-contrast imaging (SR-PCI) is a newly developed imaging modality. It uses phase-contrast information, developed by the redistribution of light intensity when penetrating tissues, and depends on the refractive effect of X-ray in sample. According to the refractive index formula, n = 1 − δ + iβ, where δ = r_e_ρ_e_λ^2^/2π and β = μλ/4π; δ is correlated with the phase and β is correlated with the absorption coefficient μ. With C2H4, for example, the phase-correlated index δ of 25 keV X-rays (λ = 0.496 A°) is 3.5 × 10^−7^, yet the absorption-correlated index β is 8.1 × 10^−11^. δ is higher than β by about 3 orders of magnitude, and the phase contrast can reach 0.0003 g cm^-3^. So in theory, the contrast of light elements can reach 1000 times that of conventional absorption imaging^19^.

The principle of synchrotron-radiation X-ray phase-contrast imaging is based on Fresnel diffraction theory. The experiment setup, when using a synchrotron radiation light source, is simple, requiring only a double-crystal monochromator, a sample stage and a detector. Figure 1 shows the real photo of the experiment setup^15^,^18^.

From the advantages mentioned above, synchrotron-radiation phase-contrast imaging has broad prospects in domains such as biomedicine and material science. *In vitro* high-resolution SR-PCI can be used for imaging breast, lung, liver and kidney tissue, and achieves excellent soft-tissue contrast without the use of enhancement agents^20^. We have successfully used it for esophageal imaging. Yet the use of SR-PCI has not been reported for the esophagogastric junction.

We used the BL13W1 beamline at the Shanghai Synchrotron Radiation Facility (SSRF), to investigate the use of phase-contrast X-ray CT imaging for the esophagogastric junction *In vitro*. In addition, we explored the ability of this imaging modality to demonstrate the fine structure in the esophagogastric junction wall layering.

## Results

**Figure 2:**
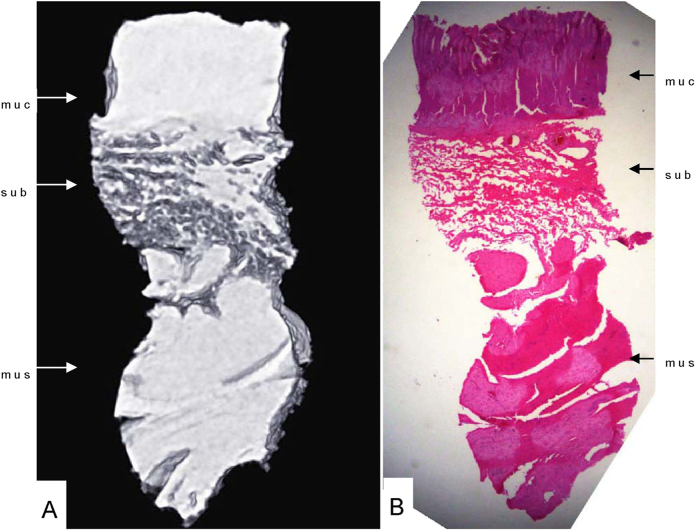
Horizontal scan (A) and histology staining of the esophagogastric junction (B). The normal esophagogastric junction wall was clearly depicted with Phase-contrast X-ray CT including the mucous, submucosa and muscular layers (**A**). muc: mucous layer; sub: submucosa layer; mus: muscular layer.

**Figure 3:**
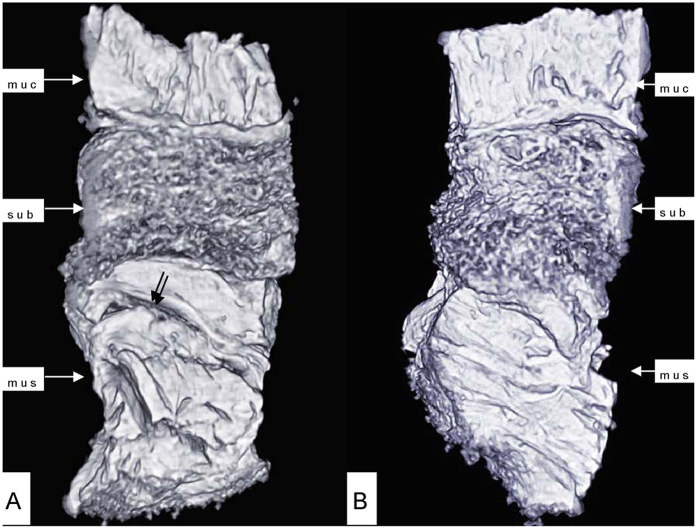
3D image of the esophagogastric junction. The shape of the mucous and muscular layers was complete. The boundary between the mucous and submucosa layers was distinct, and the same as the muscular and submucosa layers. The surface of the esophagogastric junction was displayed clearly. Crevice was revealed (double arrow in **A**).

**Figure 4:**
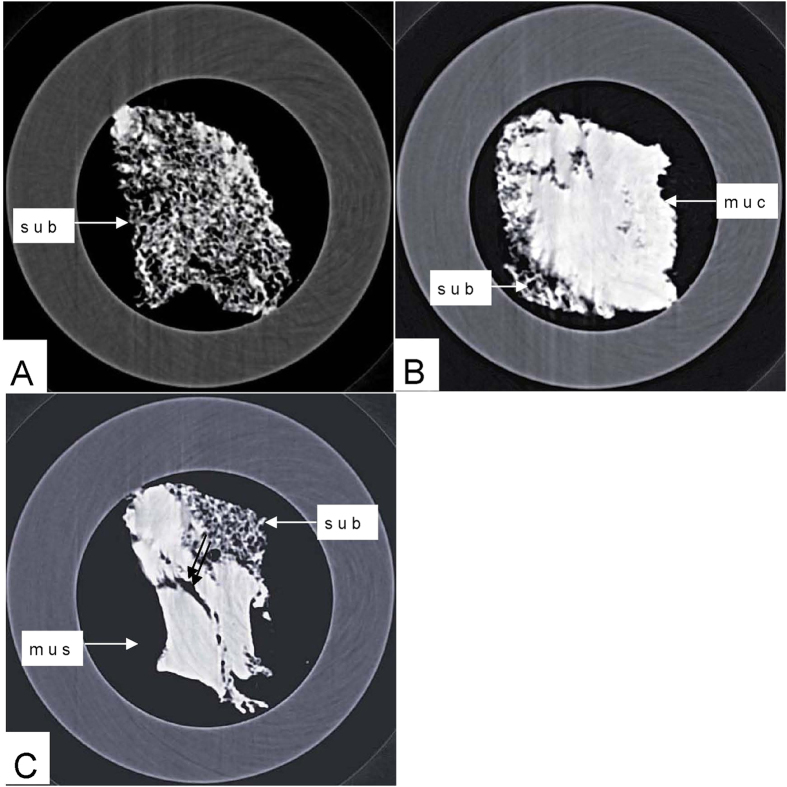
Longitudinal scan of the esophagogastric junction. Submucosa layer was displayed honeycomb appearance (**A**). The mucous and muscular layers were relatively close-grained (**B**,**C**). Some strips of tubular low-densuty areas in muscular layer were crevices (double arrow in **C**).

**Figure 5:**
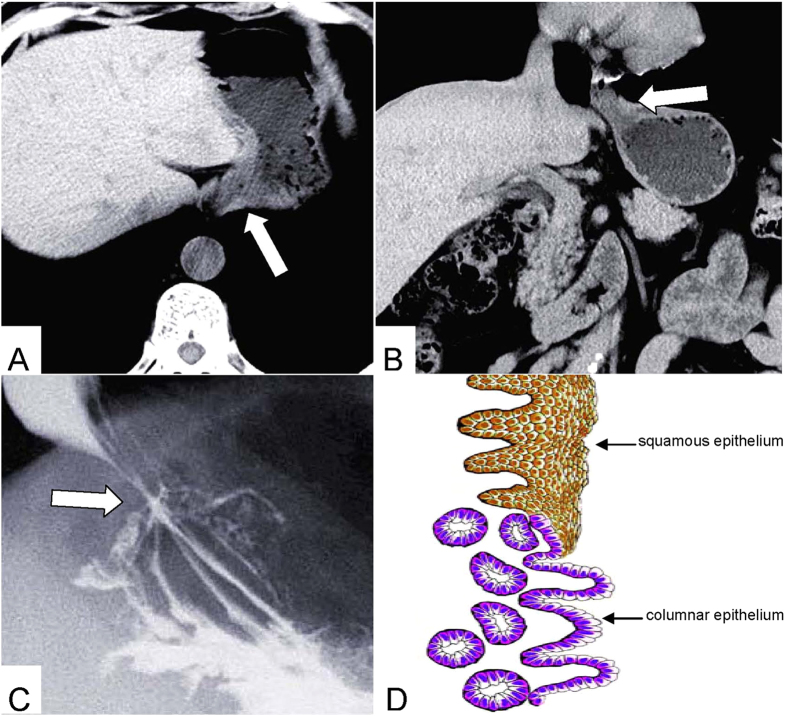
CT axial view image (**A**) coronal view image (**B**) Gas-barium double-contrast X-ray image (**C**) and sketch map (**D**) of the esophagogastric junction: (**A**,**B**) CT demonstrates the esophagogastric junction and the relation with adjacent tissues(the empty arrow). (**C**) Mucosa shows stellate high-density shadow (the empty arrow).

Phase-contrast X-ray CT imaging by synchrotron-radiation broadband monochromatic light clearly depicted the normal esophagogastric junction wall, including the mucous, submucosa and muscular layers (Figs 2 and 3). The mucous and muscular layers were clearly separated by a loose submucosa layer, with a honeycomb appearance (Fig. 4). The shape of the mucous and muscular layers was complete. The boundary between the mucous and submucosa layers was distinct, and the same as the muscular and submucosa layers. The surface of the esophagogastric junction was displayed clearly through 3D reconstruction (Fig. 3). We found many strips of tubular low-density areas in the muscular layer, which were crevices that might be caused by fiber contraction during fixation or open-air drying (Fig. 4). For comparison, we show a gas-barium double contrast X-ray image and CT image of esophagogastric junction tissue from the same patients (Fig. 5). The gas-barium double contrast X-ray image showed the mucosa, displaying stellate high-density shadows (caused by the barium sulfate coating on the esophagogastric junction mucosa surface), as well as gas filling the esophagogastric junction lumen. CT revealed the esophagogastric junction wall in relation to adjacent tissues. However, both techniques combined could not discriminate the layers of the esophagogastric junction wall.

## Discussion

Our phase-contrast X-ray CT imaging of the esophagogastric junction specimens clearly revealed the three-layer structure of the esophagogastric junction wall, comprised of the mucous, submucosa and muscular layers. In particular, the submucosa layer was distinctly different from the mucous and muscular layers and had a honeycomb appearance. The shape of the mucous and muscular layers was intact. The boundary between the mucous and submucosa layers was distinct, as was the border of the muscular and submucosa layers. The surface of the esophagogastric junction was displayed clearly through 3D reconstruction. All of these results can provide an imaging basis for the esophagogastric junction wall and might be helpful in the diagnosis of esophagogastric junction lesion, especially for the early adenocarcinoma. According to the tumor staging by the Union for International Cancer Control^19^, primary esophagogastric junction adenocarcinoma is classified into 6 stages, as shown in Table 1. When tumor invaded the submucosa (T1), the morphologic features of the submucosa change, and with muscularis propria or adventitia invasion (T2 or T3), the total submucosa is absent. Therefore, phase-contrast CT imaging can provide useful information for qualitative diagnosis of esophagogastric junction tumors, and the clarity of the image could allow for tumor staging.

With improvements in image resolution with phase-contrast CT imaging, the possibility of using X-rays to analyze tiny structures of the esophagogastric junction wall would be possible with the high flux density of 3^rd^ generation synchrotron radiation. The technology will provide useful information (the esophagogastric junction wall layering) for the imaging of the esophagogastric junction wall. In our experiment, phase-contrast X-ray CT imaging also showed mucosa. However, our specimens were fixed and air-dried, so the shape differed from the original morphologic features.

Our study has several limitations^17^. First, our specimens were fixed and air-dried, which led to water content loss, so some tissues showed differences *in vivo* and some useful information might be lost. This imaging technique still cannot be used *in vivo*. We designed this *in vitro* study in order to show the potential for future *in vivo* use. Recently, however, a Talbot-type imaging interferometer raised the possibility of yielding quantitative differential phase-contrast images with conventional X-ray tubes^21^. It is highly probable that phase-contrast X-ray imaging can be applied to clinical trials in the near future. Second, phase-contrast field of view is too narrow to adapt to large sample. With the use of a monolithic X-ray interferometer made from a silicon ingot, the maximum achievable image size is limited by its diameter. The Talbot-type imaging interferometer is currently being developed to obtain a larger field of view and might resolve this problem^21^.

In summary, phase-contrast CT imaging can clearly depict the three-layer structure of the the esophagogastric junction wall without the need for a contrast agent and can display the mucous plane without the need for barium sulfate. This technique is currently not applicable in clinical practice. We only describe a new method using phase-contrast x-ray CT that has a high sensitivity and specificity for noninvasive detection of the esophagogastric junction. The method has a high clinical potential. Once developed into a clinical routine^22^, this x-ray phase-contrast imaging methodology could have a great impact on diagnosis and treatment in patients with esophagogastric junction lesion, especially for the early adenocarcinoma.

## Methods

### Specimen preparation

We sent three samples of inferior-segment esophageal carcinoma tissue in which containing the OGJ to the Pathology Department at Shantou University Medical College for pathological examination. The esophageal carcinoma tissue was washed with 0.9% sodium chloride repeatedly to remove the coated mucus and blood, then immersed in 10% formalin solution for 1 day. Three specimens, normal esophagogastric junction wall (with mucous, submucosa, muscular and serosa layers) were extracted from the whole sample. The normal tissues, away from carcinoma tissue, were horizontally cut into strips of 10 × 4 × 3 mm. Then the 3 prepared specimens were immersed in 10% formalin for an additional 2 days. The specimens were fixed, air-dried at room temperature, placed into plastic tubes for imaging. Participants who provided samples gave their written informed consents for use of the tissue (These were also the participants who involved in the X-ray imaging and CT scanning). The methods were carried out in accordance with the approved guidelines. Our research has been approved by Ethical Committee of Shantou University Medical College.

### Phase-contrast X-ray CT imaging

Synchrotron-radiation phase-contrast X-ray CT of esophagogastric junction specimens involved use of the beamline BL13W at the SSRF. The synchrotron radiation source for the BL13W is a hybrid-type wiggler with periodic length was 14 cm and a period number of 8. The fundamental radiation covered from 8.0 to 72.5 keV energy by tuning the gap from 17 to 35 mm. A fixed-exit double-crystal cryogenic-cooling monochromator was placed 28 m from the source point. The monochromator crystal was a combination of a Si(111) orientation crystal and a Si(311) orientation crystal. A high-precision sample stage, allowing for translation along all of the three-space directions with resolution >1 micron, and rotation in all 3 axes was used for positioning the sample and rotating the sample axis perpendicular to the beam. The total acceptance angle was 1.5 mrad in the horizontal direction and 0.2 mrad in the vertical direction. In our experiment, the electron energy was adjusted to 15 keV.

The highly parallel and monochromatic beam was projected on the object being imaged. When X-ray beams traveled through the object, the downstream beams carry the absorption and phase shift information. After propagating a sufficient 9-cm distance, the phase shifts in the downstream beams are transformed into measurable intensity variations by Fresnel diffraction. An X-ray-sensitive CCD camera, with maximum 2048 × 2048 pixels of 3.7 × 3.7 μm, was used as a 2D detector to transform the beam into an image. During the CT data acquisition, the specimen was rotated around its cylinder axis for 180°. The number of projections was 900, with exposure time 1 s for each projection. Acquired images were reconstructed into 2D and 3D images respectively with X-TRACT (TIE-Hom phase retrieval algorithm with *γ* = 550) and Amira, and compared with histopathology findings.

### X-ray imaging and CT scanning

These patients were suggested to abdmen-thorax CT scanning, then to Gas-barium double contrast X-ray gastrointestinalgraphy of the esophagogastric junction before resection. Before the X-ray examination, some aerogenic powders are taken orally by patients. Reactions between the dry powders won’t take place until they encounter water. The esophagogastric junction will be expanded by the produced CO2 gas. After a few minutes, the patients take orally barium sulfate. The mucosa and filling phase are dynamic observed.

### Histopathology

The imaged formalin-fixed samples were dehydrated and embedded in paraffin, then cut into 4 μm-thick sections. Samples were stained with hematoxylin and eosin and examined by light microscopy.

## Additional Information

**How to cite this article**: Zhang, J. *et al.* Microdissection of Human Esophagogastric Junction Wall with Phase-contrast X-ray CT Imaging. *Sci. Rep.* **5**, 13831; doi: 10.1038/srep13831 (2015).

## Figures and Tables

**Figure 1:**
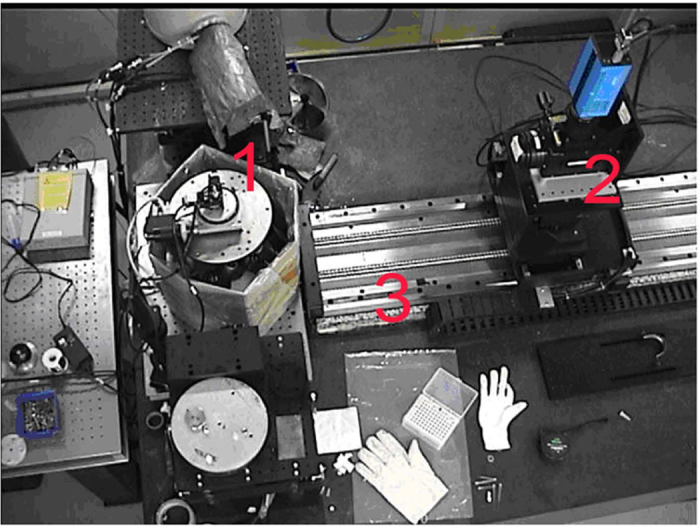
The picture of BL13W1 beamline partial facility of SSRF. 1. A multidimensional specimen table. The specimens are placed on the specimen table to rotate and specimens were obtained in different angles. 2. An X-ray CCD. It obtained specimens’ projective images with high-resolution. 3. The precise guide rail. It can control the exact distance from the CCD to the specimens.

**Table 1:** Tumor staging of esophagogastric junction adenocarcinoma.

T0	No evidence of tumor
Tis	Carcinoma *in situ*
T1	Tumor invades lamina propria or submucosa
T2	Tumor invades muscularis propria
T3	Tumor invades adventitia
T4	Tumor invades adjacent structures
